# Supplementary material for: Creating two-dimensional solid helium via diamond lattice confinement
Source: Nat Commun. 2022 Oct 11;13:5990. doi: 10.1038/s41467-022-33601-5 (PMC9553866; doi:10.1038/s41467-022-33601-5)
Supplement: Supplementary file 3 — Solar Cells Reporting Summary [file 41467_2022_33601_MOESM3_ESM.pdf]

nature research

Corresponding author(s): Tao Yang, Ji-jung Kai

Solar Cells Reporting Summary

Nature Research wishes to improve the reproducibility of the work that we publish. This form is intended for publication with all accepted papers reporting the characterization of photovoltaic devices and provides structure for consistency and transparency in reporting. Some list items might not apply to an individual manuscript, but all fields must be completed for clarity.

For further information on Nature Research policies, including our data availability policy, see Authors & Referees.

ü Experimental design

Please check: are the following details reported in the manuscript?

1. Dimensions

Item	Yes/No	Explanation
Area of the tested solar cells	☐ Yes ☒ No	Solar cells were not studied in our paper, so we didn't test solar cells.
Method used to determine the device area	☐ Yes ☒ No	Solar cells were not studied in our paper, so we didn't determine the device area.

2. Current-voltage characterization

Item	Yes/No	Explanation
Current density-voltage (J-V) plots in both forward and backward direction	☐ Yes ☒ No	Solar cells were not studied in our paper, so we didn't perform current-voltage characterization.
Voltage scan conditions<br>*For instance: scan direction, speed, dwell times*	☐ Yes ☒ No	Solar cells were not studied in our paper, so we didn't perform current-voltage characterization.
Test environment<br>*For instance: characterization temperature, in air or in glove box*	☐ Yes ☒ No	Solar cells were not studied in our paper, so we didn't test solar cells.
Protocol for preconditioning of the device before its characterization	☐ Yes ☒ No	Solar cells were not studied in our paper, so we didn't have Protocol for preconditioning of the device.
Stability of the J-V characteristic<br>*Verified with time evolution of the maximum power point or with the photocurrent at maximum power point; see ref. 7 for details.*	☐ Yes ☒ No	Solar cells were not studied in our paper, so we didn't perform current-voltage characterization.

3. Hysteresis or any other unusual behaviour

Item	Yes/No	Explanation
Description of the unusual behaviour observed during the characterization	☐ Yes ☒ No	Solar cells were not studied in our paper, so we didn't test solar cells.
Related experimental data	☐ Yes ☒ No	Solar cells were not studied in our paper, so we didn't test solar cells.

4. Efficiency

Item	Yes/No	Explanation
External quantum efficiency (EQE) or incident photons to current efficiency (IPCE)	☐ Yes ☒ No	Solar cells were not studied in our paper, so we didn't test the efficiency of solar cells.
A comparison between the integrated response under the standard reference spectrum and the response measure under the simulator	☐ Yes ☒ No	Solar cells were not studied in our paper, so we didn't test the efficiency of solar cells.
For tandem solar cells, the bias illumination and bias voltage used for each subcell	☐ Yes ☒ No	Solar cells were not studied in our paper, so we didn't test the efficiency of solar cells.

5. Calibration

Item	Yes/No	Explanation
Light source and reference cell or sensor used for the characterization	☐ Yes ☒ No	Solar cells were not studied in our paper, so we didn't have the light source or reference cell.
Confirmation that the reference cell was calibrated and certified	☐ Yes ☒ No	Solar cells were not studied in our paper, so we didn't have the reference cell.

Calculation of spectral mismatch between the reference cell and the devices under test

- [ ] Yes
- [x] No

Solar cells were not studied in our paper, so we didn't have the reference cell.

6. Mask/aperture

Size of the mask/aperture used during testing

- [ ] Yes
- [x] No

Solar cells were not studied in our paper, so we didn't have the mask or aperture.

Variation of the measured short-circuit current density with the mask/aperture area

- [ ] Yes
- [x] No

Solar cells were not studied in our paper, so we didn't measure the short-circuit current density.

7. Performance certification

Identity of the independent certification laboratory that confirmed the photovoltaic performance

- [ ] Yes
- [x] No

Solar cells were not studied in our paper, so we didn't have the performance certification.

A copy of any certificate(s)
Provide in Supplementary Information

- [ ] Yes
- [x] No

Solar cells were not studied in our paper, so we didn't have the performance certification.

8. Statistics

Number of solar cells tested

- [ ] Yes
- [x] No

Solar cells were not studied in our paper, so we didn't test solar cells.

Statistical analysis of the device performance

- [ ] Yes
- [x] No

Solar cells were not studied in our paper, so we didn't test solar cells.

9. Long-term stability analysis

Type of analysis, bias conditions and environmental conditions
For instance: illumination type, temperature, atmosphere humidity, encapsulation method, preconditioning temperature

- [ ] Yes
- [x] No

Solar cells were not studied in our paper, so we didn't perform long-term stability analysis.
