# Supplementary material for: Creating two-dimensional solid helium via diamond lattice confinement
Source: Nat Commun. 2022 Oct 11;13:5990. doi: 10.1038/s41467-022-33601-5 (PMC9553866; doi:10.1038/s41467-022-33601-5)
Supplement: Supplementary file 4 — Description of Additional Supplementary Files [file 41467_2022_33601_MOESM4_ESM.pdf]

Description of Additional Supplementary Files

File Name: Supplementary Data 1
Description: A cif file containing atomic positions of the two-dimensional solid helium stabilized by diamond lattice
