# Supplementary material for: Creating two-dimensional solid helium via diamond lattice confinement
Source: Nat Commun. 2022 Oct 11;13:5990. doi: 10.1038/s41467-022-33601-5 (PMC9553866; doi:10.1038/s41467-022-33601-5)
Supplement: Supplementary file 5 — Supplementary information [file 41467_2022_33601_MOESM5_ESM.pdf]

Supplementary Information for

Creating two-dimensional solid helium via diamond lattice confinement

Weitong Lin et al.

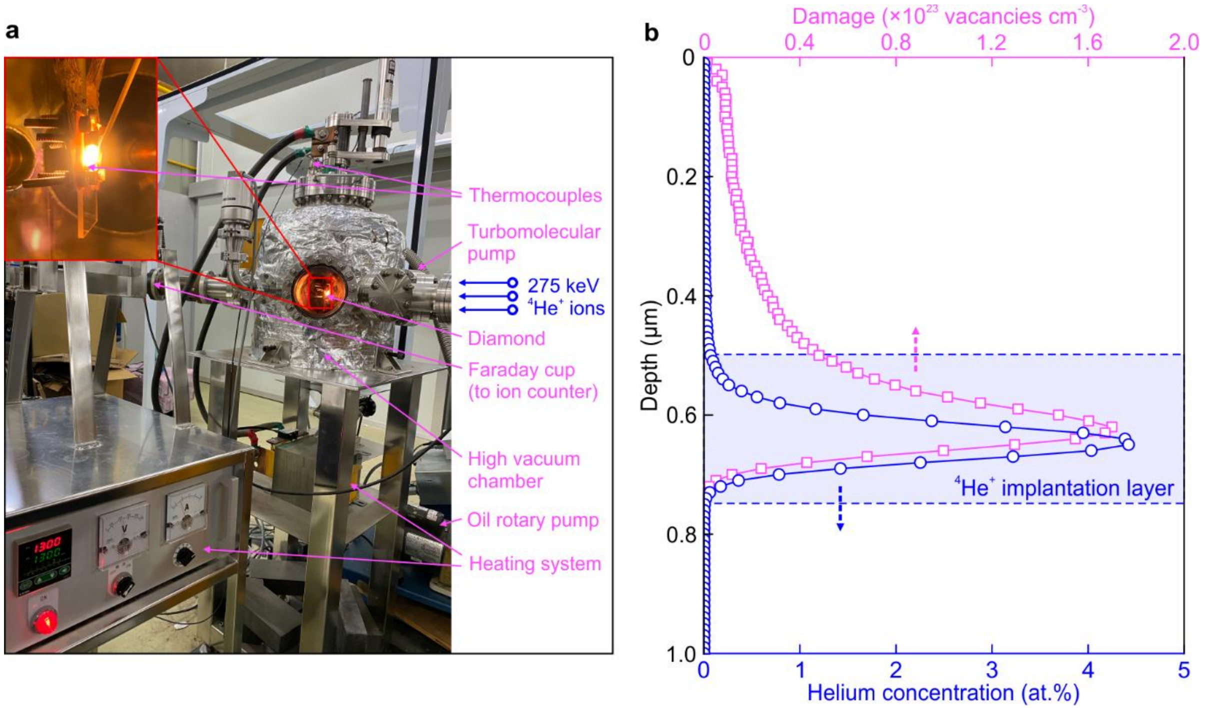

Supplementary Fig. 1 | Ion implantation setup and predictions. a, Photo of high-temperature ion implantation setup, including $^{4}He^{+}$ ions beam, Faraday cup, high vacuum chamber, oil rotary pump, turbomolecular pump, Pt/Pt-Rh thermocouples, and heating system. **b,** Predictions of irradiation damage and helium distributions along the cross-section direction in diamond subjected to helium ion implantation. Source data are provided as a Source Data file.

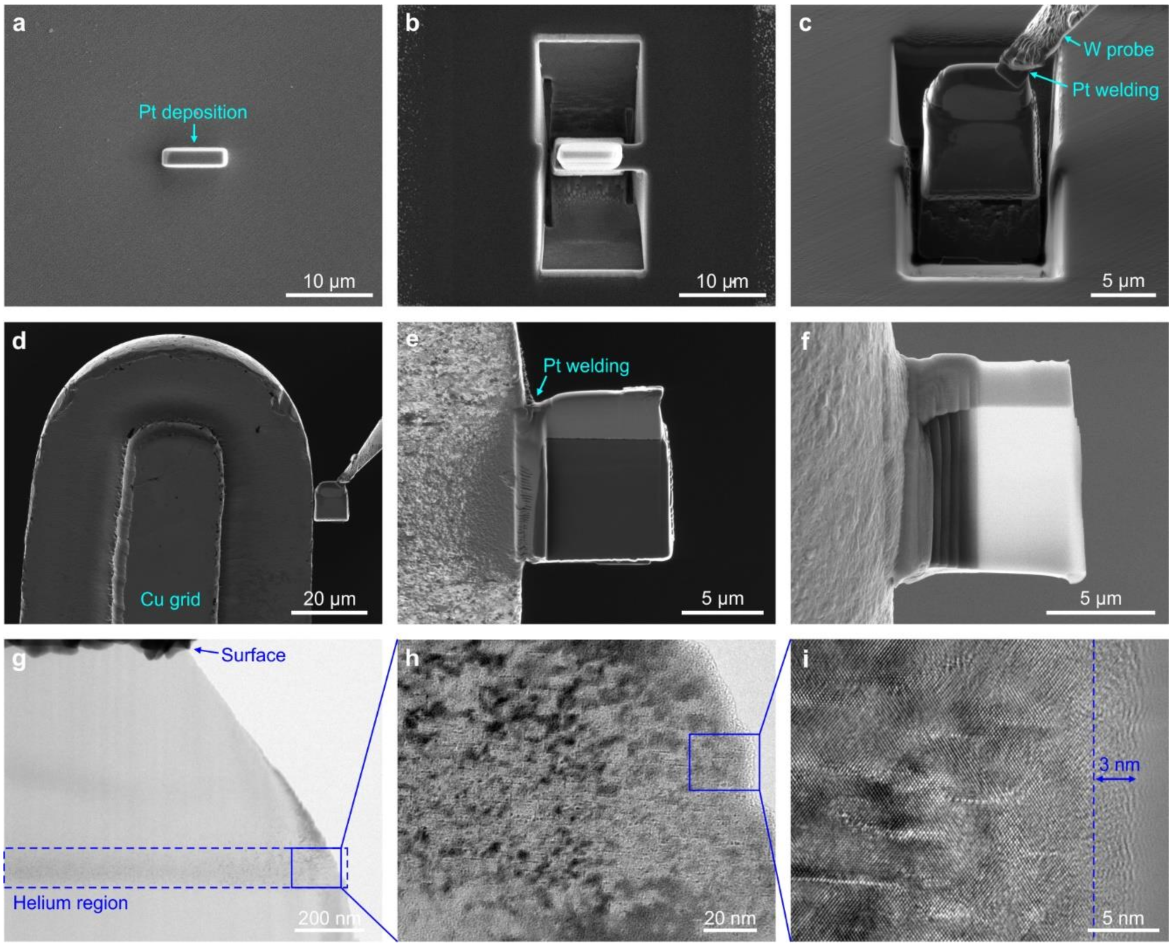

Supplementary Fig. 2 | Preparation process of TEM specimens. a, SEM image shows Pt deposition on the diamond surface. **b-e,** SEM images show diamond lamella lift-out and mount to a copper grid. **f**, SEM image shows the TEM specimen after FIB thinning. **g, h,** Bright-field TEM images show a typical diamond specimen composed of the helium region. **i,** HRTEM image shows the high-quality TEM specimen with a 3-nm-thick amorphous layer.

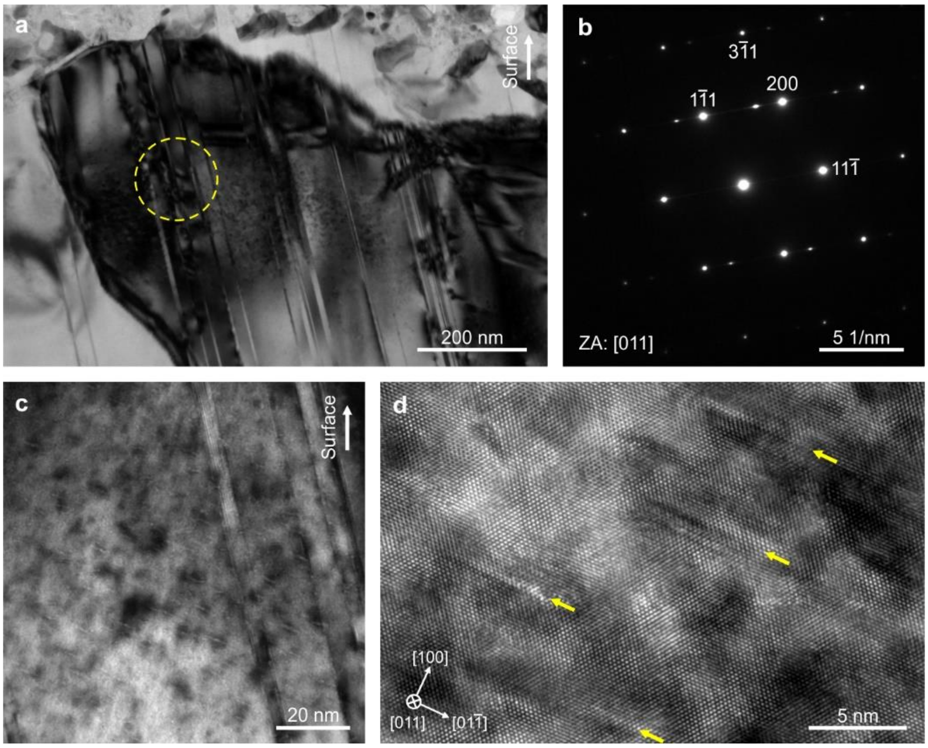

Supplementary Fig. 3 | Crystallographic orientation of helium platelets in diamonds. a, Bright-field TEM image shows a polycrystalline diamond subjected to 275 keV $^{4}He^{+}$ ion implantation at 1,573 K. The total fluence was 1.0×10^{16} cm^{-2}, and the beam flux was 9.4×10^{11} ions cm^{-2} s^{-1}. Some lamellar {111} nanotwins were also observed. **b**, Selected area electron diffraction image taken from the region indicated by the yellow circle in (**a**), suggesting the [$3\bar{1}1$] crystalline orientation of the diamond grain with respect to the ion implantation direction. The zone axis (ZA) is [011]. **c**, Fresnel contrast TEM image demonstrates helium platelets in the under-focus condition. **d**, HRTEM image shows helium platelets highlighted by yellow arrows.

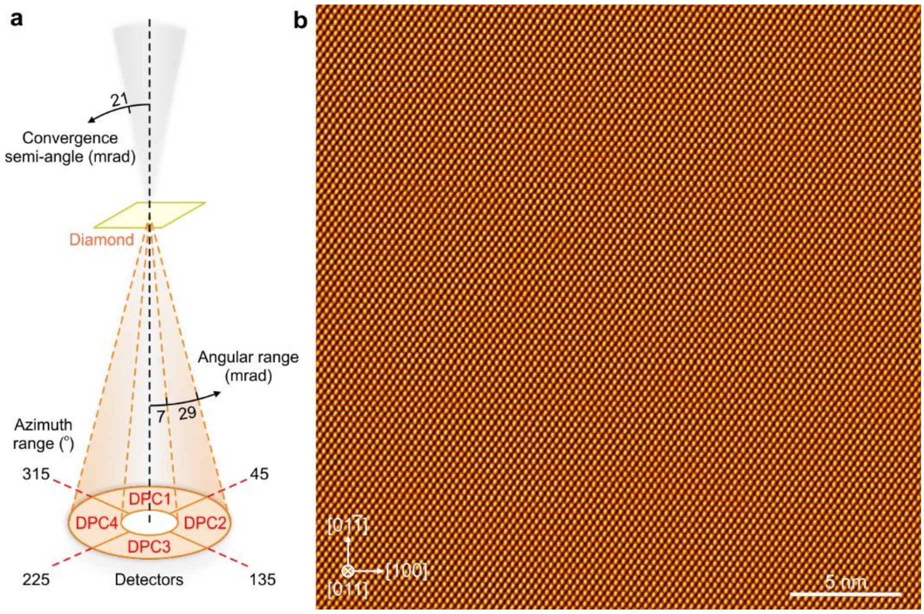

Supplementary Fig. 4 | iDPC STEM characterization. a, Schematic illustration of the iDPC STEM setup. **b,** iDPC STEM image shows the pristine diamond. The zone axis is [011].

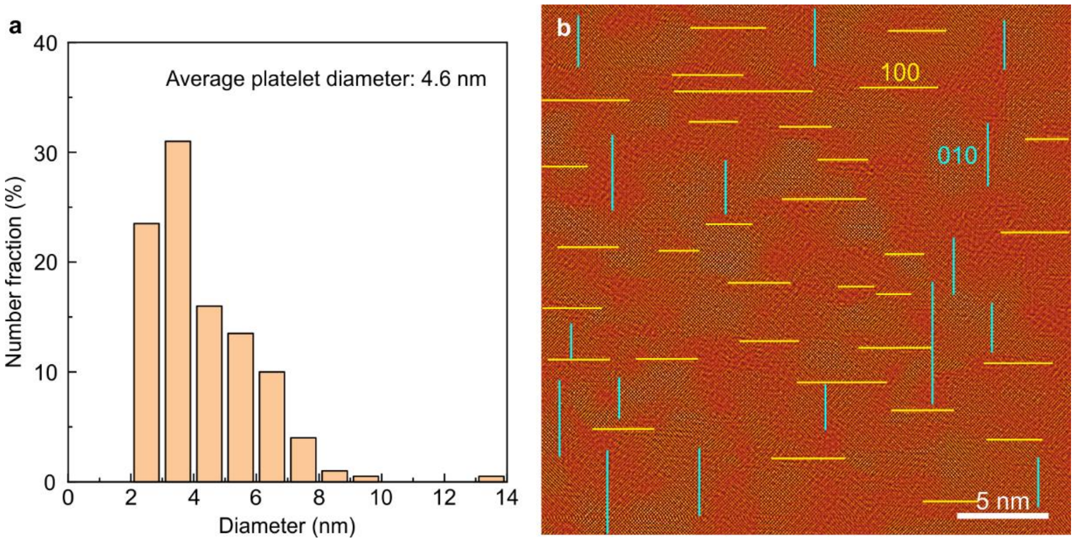

Supplementary Fig. 5 | Diameter distribution and habit plane of helium platelets in diamond. a, The diameter distribution of helium platelets in diamond. Source data are provided as a Source Data file. **b,** iDPC STEM image under the [001] zone axis, where (100) and (010) helium platelets are marked by yellow and blue lines, respectively.

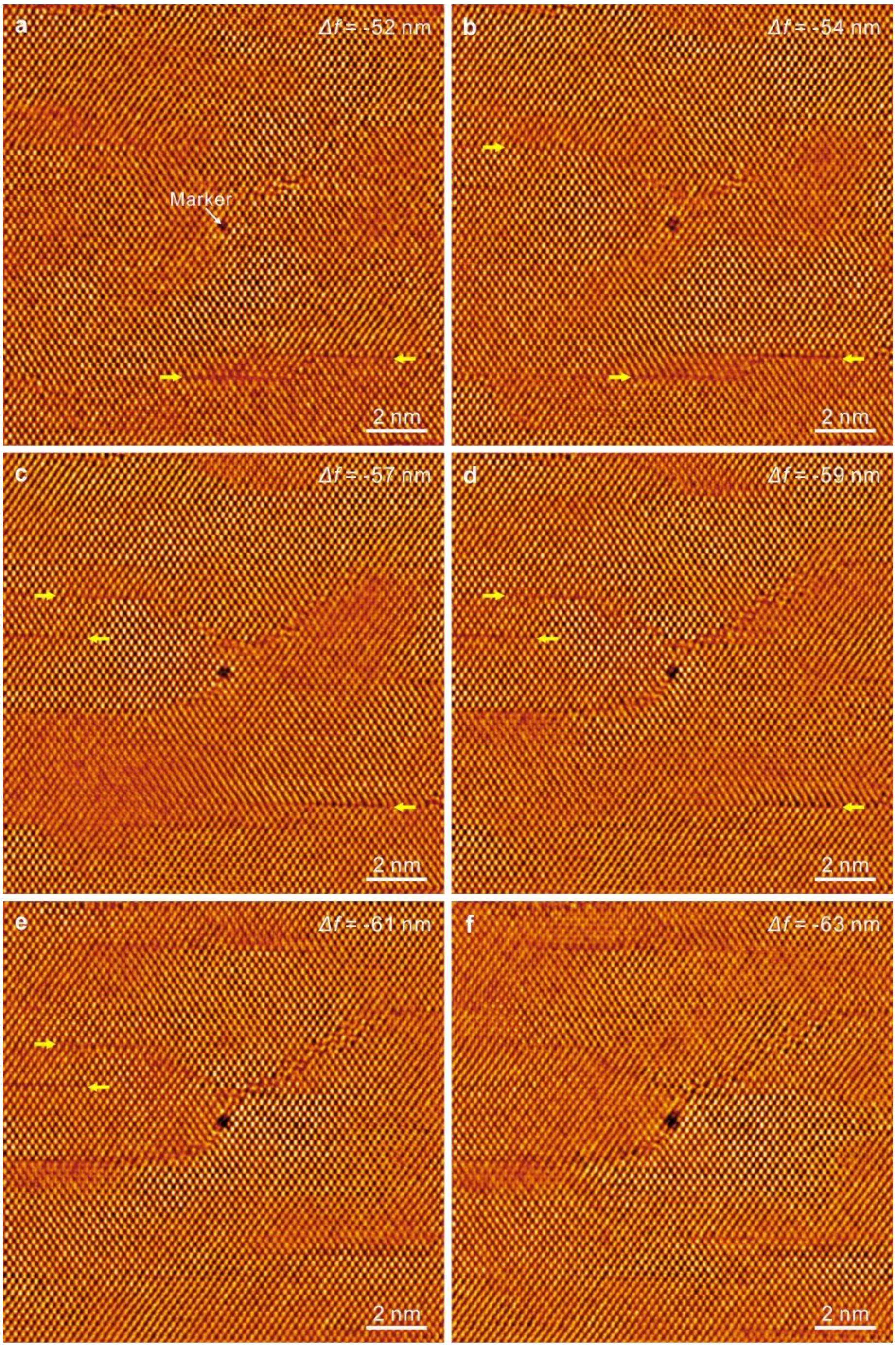

Supplementary Fig. 6 | Depth-sectioning iDPC STEM imaging. a-f, A through-focal series of iDPC STEM images show helium platelets in the diamond specimen, some of which are highlighted by yellow arrows, with the defocus of (**a**) -52, (**b**) -54, (**c**) -57, (**d**) -59, (**e**) -61, and (**f**) -63 nm. To precisely observe the same region of the TEM specimen, a hole was artificially constructed as a marker in the centre of the image by focusing the electron beam.

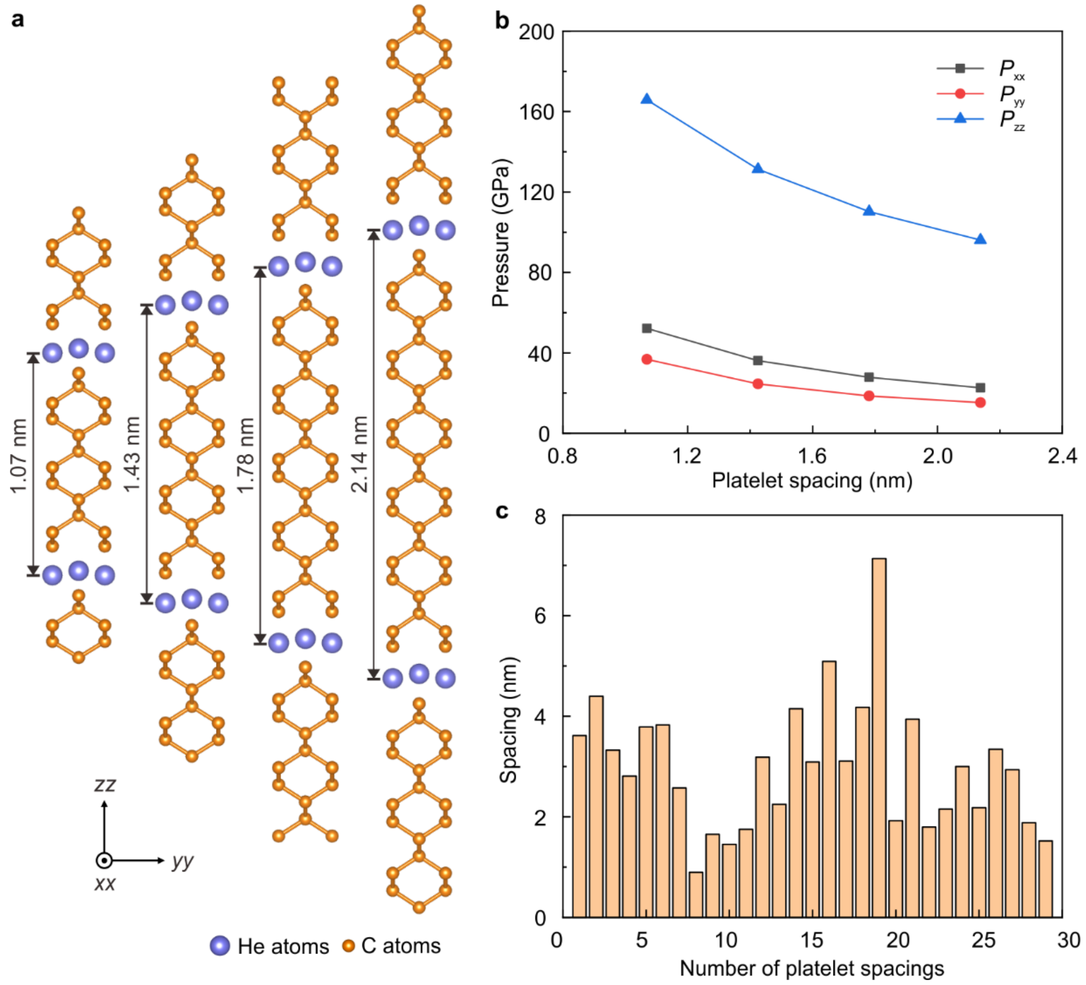

Supplementary Fig. 7 | Helium pressure and platelet spacing. a, Atomic structural models of {100} two-dimensional solid helium with different platelet spacings. **b,** Pressure inside four models in (**a**) predicted by DFT calculations. **c,** Spacings between helium platelets measured by iDPC STEM imaging. Source data are provided as a Source Data file.

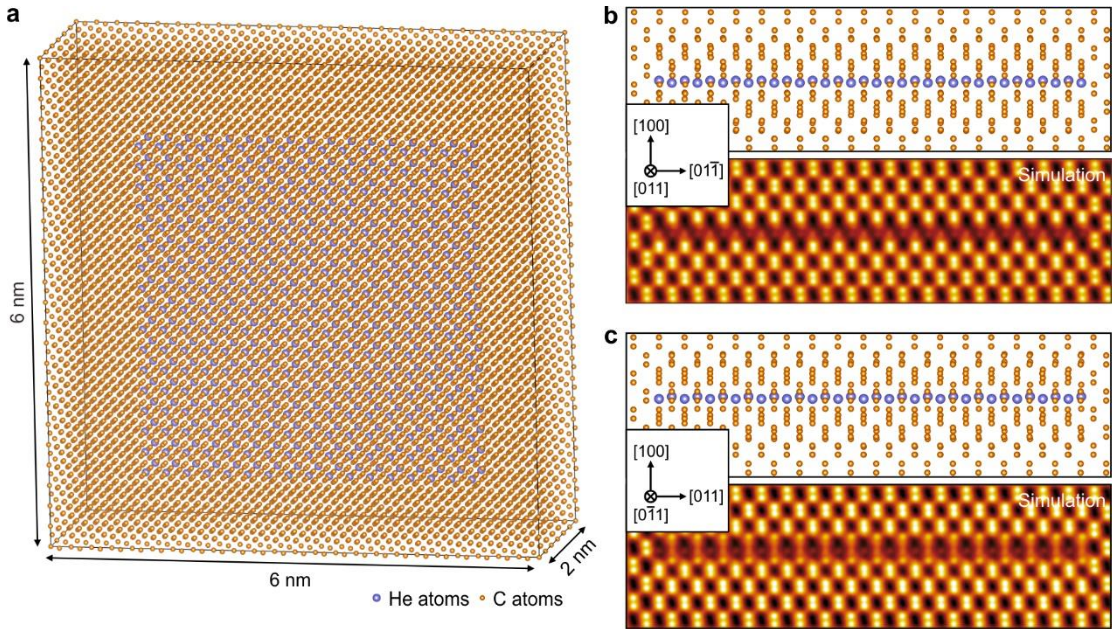

Supplementary Fig. 8 | iDPC STEM image simulation. a, Three-dimensional sample data for image simulations. **b, c,** Simulated iDPC STEM images of two-dimensional solid helium in diamonds oriented along (**b**) [011] and (**c**) $[0\bar{1}1]$ zone axis. Despite the relatively small atomic model cannot completely represent actual TEM specimens, the simulated images exhibit similar atomic arrangements with experimental results that can support the findings.

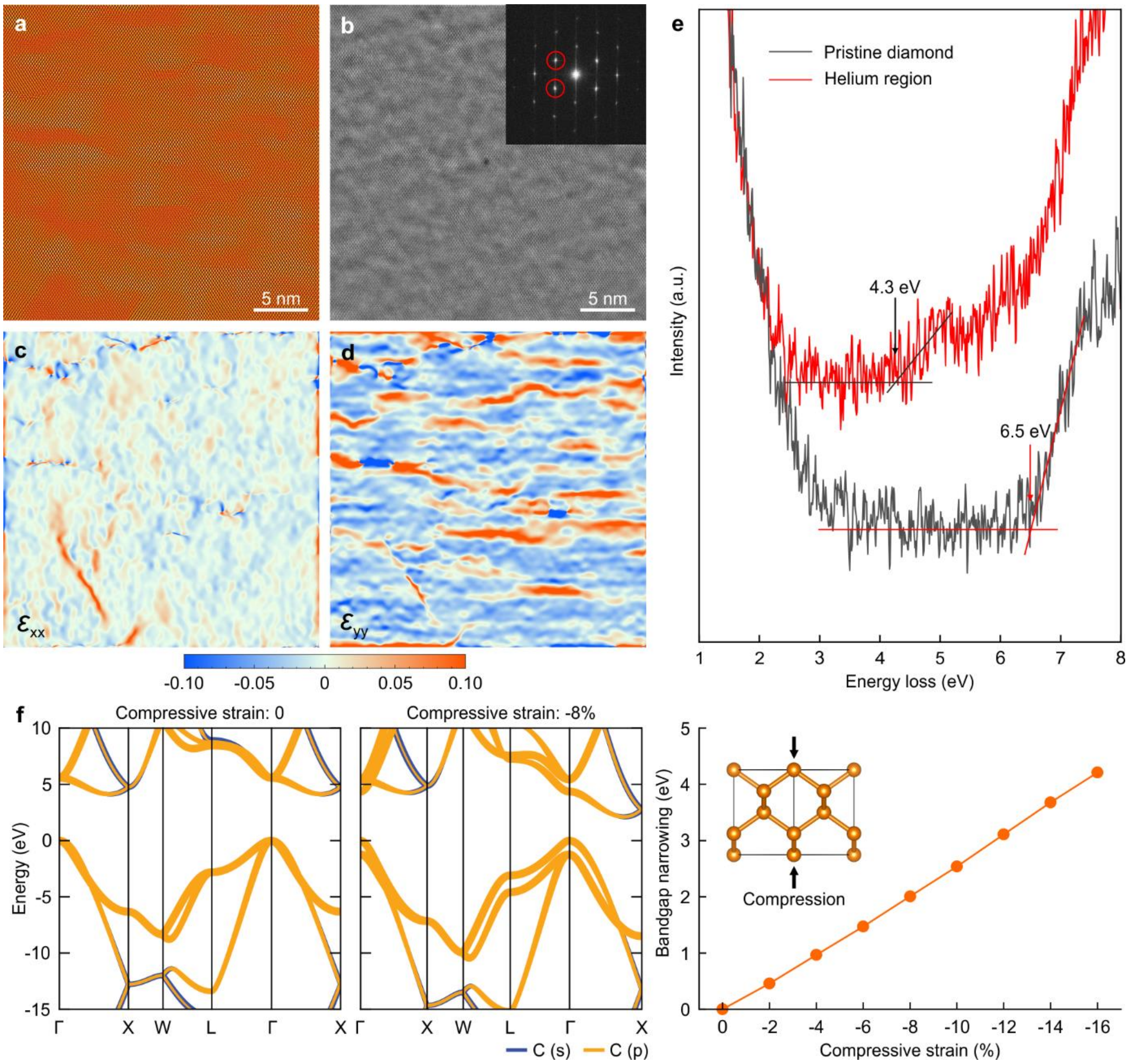

Supplementary Fig. 9 | Bandgap tuning of diamond. a, iDPC STEM image of the helium region. **b,** Corresponding ADF STEM image. Inset: the fast Fourier transform pattern obtained from the entire image. **c,** Horizontal normal strain (ε_{xx}) map. **d,** Vertical normal strain (ε_{yy}) map. **e,** Off-axis STEM-EELS spectra obtained at the $1\bar{1}1$ Bragg diffraction disk. The bandgap onsets of the helium region and the pristine diamond are determined to be 4.3 and 6.5 eV, respectively. The bandgap of the pristine diamond is a little larger than the indirect bandgap measured by optical methods[1] (that is, 5.5 eV) because the off-axis EELS mode increases the momentum transfer $\boldsymbol{q}$ value. **f,** DFT predictions show bandgap narrowing in the <100>-oriented diamond subjected to compressive strains. Source data are provided as a Source Data file.

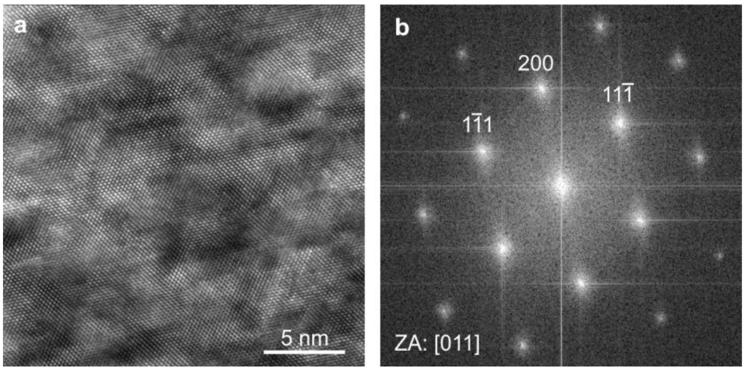

Supplementary Fig. 10 | TEM characterization of diamond implanted at 1173 K. a, HRTEM image of the helium implantation peak region. **b,** Fast Fourier transform image taken from the region, revealing that amorphization and graphitization were suppressed. The zone axis (ZA) is [011].

Supplementary Reference

1. Zaitsev, A. M. *Optical Properties of Diamond: A Data Handbook*. (Springer Science & Business Media, 2013).
